# Examination of the Communication Environment When Applying Removable Dentures as Wearable Devices

**DOI:** 10.7759/cureus.97531

**Published:** 2025-11-23

**Authors:** Yoshihiro Iwata, Yuna Yanagizono, Takashi Iida, Yuusaku Ashida, Yuuta Yamada, Takashi Asano, Hiroshi Suzuki, Takuma Tsuge, Osamu Komiyama, Kiichi Niitsu

**Affiliations:** 1 Department of Prosthodontics and Oral Rehabilitation, Nihon University School of Dentistry at Matsudo, Chiba, JPN; 2 Graduate School of Informatics, Kyoto University, Kyoto, JPN

**Keywords:** communication environment, ic tags, real-time monitoring, removable dentures, wearable devices

## Abstract

Introduction

Using removable dentures as wearable devices will be beneficial for patients at home. This study aimed to clarify the communication environment when applying integrated circuit (IC) tags to removable dentures, considering the effects of saliva, encapsulation depth, and encapsulation materials.

Methods

Fifteen specimens were made for each encapsulating depth (4.5 mm, 5.0 mm, 5.5 mm, 6.0 mm), and the IC tags were encapsulated in various dental materials. Reading and writing were performed on the IC tags, and the number of test specimens that could communicate at each depth before and after immersion was calculated.

Results

In the control group, which was not encapsulated in dental materials, communication was feasible up to a depth of 5.5 mm in all test specimens, but communication was unfeasible at 6 mm. In all other materials, communication was feasible up to an encapsulation depth of 5.0 mm in all test specimens, regardless of immersion in artificial saliva, but communication was unfeasible at depths of 5.5 mm or more.

Conclusions

The encapsulation depth of the IC tag and the dental materials used for encapsulating the tag affected communication capability, but immersion in artificial saliva did not. The possibility of using removable dentures as wearable devices is thus suggested.

## Introduction

Real-time monitoring of physiological status (e.g., heart rate, blood pressure) provides useful information when treating a patient, particularly for those receiving care at home [1]. Such monitoring can also detect sudden changes in the condition of the patient [2].

In the field of dentistry, since the onset of the COVID-19 pandemic in early 2020, many elderly patients have hesitated to visit dental clinics due to concerns about infection, resulting in an increased demand for home-based dental care. However, in early August 2020, the World Health Organization issued a statement emphasizing the importance of maintaining oral health during the pandemic, while recommending the postponement of routine, non-urgent oral examinations, dental cleanings, and preventive care. Against this backdrop, the spread of COVID-19 accelerated new initiatives in healthcare, including the expanded use of online medical consultations. In dentistry as well, it is anticipated that future practice will continue to require strategies and treatment modalities designed with the assumption of potential new infectious disease outbreaks [3].

Previously, for patients receiving care at home, medical professionals needed to visit the home to provide medical care, placing large demands on personnel and time. In Norway, this burden is compounded by workforce shortages. Currently, there is an estimated 18% shortage of registered nurses in home care and nursing homes, primarily due to unfilled positions and extended absences. Furthermore, Statistics Norway projects that by 2030, the country will face an under-coverage of approximately 41,500 assistant nurses and 13,000 registered nurses, underscoring the urgent need to address staffing challenges in home care services [4]. The majority of home-visit nursing agencies in Japan are small in size with fewer than five full-time equivalent nurses. Due to this limited staffing, these agencies are generally unable to provide 24-hour home-visit nursing services, which restricts their capacity to deliver end-of-life care [5]. With the growing elderly population, the demand for medical professionals is expected to increase significantly in the coming years. By the late 2030s, Japan will require approximately 14,000 additional full-time equivalent home care physicians, or 28,000 by head count, which represents a 1.72-fold and 1.64-fold increase, respectively, compared to the numbers in 2020 [6]. Enabling real-time monitoring of physiological information from remote locations using wearable devices will help solve such issues, as physiological measurements have traditionally been performed by doctors and other medical professionals in the home.

Currently, various types of wearable devices can be used for monitoring physiological information, including wristwatch-style devices and mouthguard-type devices [7,8]. If removable dentures used in dental treatment could be used as wearable devices, real-time monitoring could be performed without the need for external monitoring equipment by incorporating sensors in the removable dentures, since patients who wear removable dentures would wear them all the time except when sleeping. Further, as with regular removable dentures, resistance and discomfort felt when wearing them can be avoided if the dentist makes sufficient adjustments. In addition, when applying a mouthguard-type appliance for patient monitoring, it is necessary to fabricate a new device specifically for that purpose, which the patient must then wear. In contrast, when utilizing a removable denture, no additional device is required; the integrated circuit (IC) tag can simply be attached to the existing denture, thereby minimizing the burden on the patient and enhancing acceptability. This is particularly relevant for patients who already wear dentures, as the application of a mouthguard-type appliance in such cases would require the simultaneous use of at least two intraoral devices, potentially increasing discomfort and reducing compliance. Therefore, the application of removable dentures as wearable devices holds significant clinical value.

Research and technological development into continuous monitoring of physiological information is being actively conducted both in Japan and overseas [9,10]. As a means of achieving continuous blood glucose monitoring, Niitsu et al. introduced a bioelectricity-generating element that generates electricity from glucose in bodily fluids. They also developed a world-first "integrated power generating and sensing integrated sensor technology" that continuously generates electricity from sugar in lacrimal fluid and transmits blood glucose data with high temporal resolution. This allowed successful testing of a contact lens-type blood glucose monitor that does not require a glasses-type terminal [11,12]. However, no research to date appears to have been conducted into using removable dentures as wearable devices.

If removable dentures were to be used as a wearable device, the communication environment would be the oral environment, and the effects of constituents such as saliva may deteriorate the communication conditions, such as reducing the communication distance or even preventing communication. Previous study by Fukuda et al. suggested that the communication conditions (damage to IC tags, rain or liquid leakage) affect the ability to utilize IC tags [13]. In addition, the encapsulation depth of the sensor and the material used for encapsulation would also affect communication conditions. Therefore, when designing removable dentures as wearable devices, the oral environment needs to be reproduced to allow consideration of issues such as communication distance, encapsulation depth and encapsulating materials. Accordingly, the purpose of this study was to clarify the communication environment when applying IC tags to removable dentures, considering the effects of saliva, encapsulation depth, and encapsulation materials, as a step toward applying removable dentures as wearable devices.

Measures to address oral frailty are necessary to extend the healthy lifespan of elderly individuals and promote the advent of a society in which elderly generations can play active roles [14]. Previous research on frailty has shown relationships between the number of remaining teeth, chewing ability, and nutritional intake in elderly people, and has reported the importance of oral function, including preserving remaining teeth and maintaining and improving chewing ability [15,16].

Moreover, in terms of the relationship between chewing ability and healthy life expectancy, differences in chewing ability have been reported to result in significant differences in healthy life expectancy across all groups aged over 65 [17]. Maintaining, restoring, and stabilizing oral functions, such as chewing ability, thus contributes to preventing frailty and extending healthy life expectancy. As mentioned earlier, removable dentures can always be worn while awake, providing a suitable wearable device for acquiring and analyzing oral data related to oral functions such as chewing ability and the ability to form a bolus for swallowing food, with the aim of maintaining, restoring, and stabilizing oral functions. The application of removable dentures as a wearable device would thus contribute to preventing frailty and extending healthy lifespan.

## Materials and methods

Test methods

**Figure 1:**
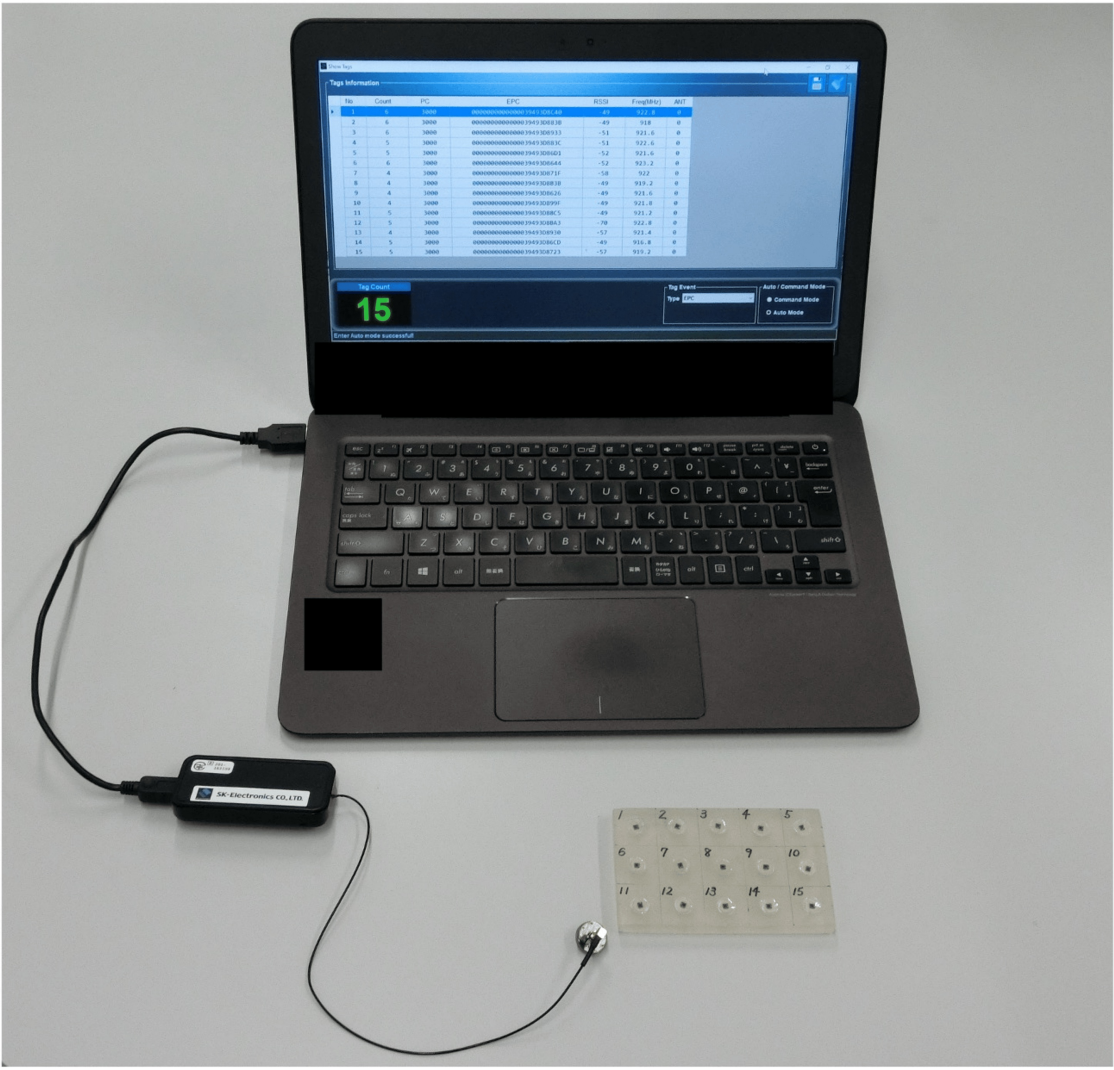
Device set-up for the experiment.

RFID IC tags (micro metal tags; SK-Electronics Co., Kyoto, Japan) were used in this study. The carrier frequency of the IC tags is in the UHF band, ranging from 860 to 960 MHz, and the communication distance, as stated by the manufacturer, is 5.0 mm. The dimensions of the IC tags are 2.2×2.5×0.91 mm. To read and write communication information, reader/writer (reader/writer antenna; SK-Electronics Co.) and software (UUG-M25A; SK-Electronics Co.) were used in this study (Figure 1).

Fabrication of test specimens

**Figure 2:**
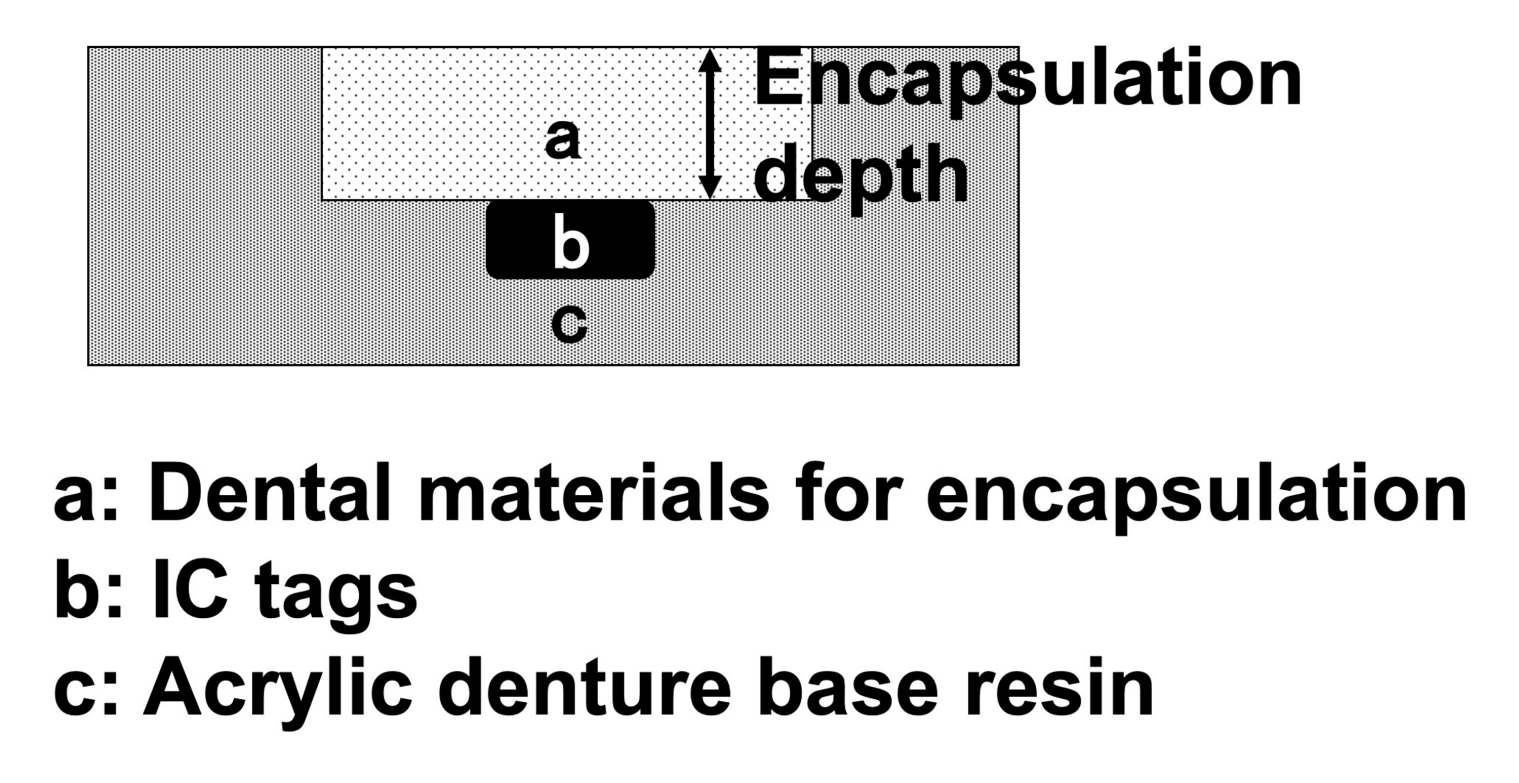
Composition of the test specimen.

A silicone master plate was fabricated using an addition-type silicone impression material (Exahiflex Tray Type; GC Corp., Tokyo, Japan). By investing silicone master plate using hard dental stone (New Plastone II; GC Corp.) stone mold was fabricated. The stone mold was invested in a dental flask (Whip Mix Co., Louisville, KY, USA) using hard dental stone (New Plastone II; GC Corp.) and allowed to set. After setting, autopolymerizing resin (Procast DSP; GC Corp.) was poured into the mold and polymerized under pressure using a hydraulic flask press (Dental Press; Morita Corp., Tokyo, Japan) at 0.2 MPa and 55 °C for 30 minutes in accordance with the instructions from the manufacturer. After polymerization, specimens were deflasked, and excess material was removed. Cavities were made at each encapsulating depth (4.5 mm, 5.0 mm, 5.5 mm, 6.0 mm), then IC tags were placed and encapsulated using the following dental materials: Unifast III Live Pink, Unifast III A2, Clearfil Majesty ES Flow, and PRG Protect Seal (Table 1). Fifteen specimens were made for each depth: 4.5 mm (n = 15), 5.0 mm (n = 15), 5.5 mm (n = 15), 6.0 mm (n = 15), respectively. The compositions of test specimens are shown in Figure 2. In addition, specimens in which the IC tags were not encapsulated in dental materials were used as a control group.

**Table 1:** Dental materials used to encapsulate IC tags.

Generic name	Material	Manufacturer
Self-curing acrylic resin for temporary inlays, crowns, bridges & repairs	UNIFAST III Live Pink	GC Corp., Tokyo, Japan
Self-curing acrylic resin for temporary inlays, crowns, bridges & repairs	UNIFAST III A2	GC Corp., Tokyo, Japan
Dental light-cured restorative	CLEARFIL MAJESTY ES FLOW	Kuraray Noritake Dental Inc., Tokyo, Japan
Temporary filling materials	PRG PROTECT SEAL	SHOFU Inc., Kyoto, Japan

Measurements

To reproduce the oral environment, artificial saliva BZ109 (Biochemazone Inc., Alberta, Canada) was used as a substitute for saliva. According to the manufacturer’s Certificate of Conformity, the main components include sodium chloride, potassium chloride, calcium chloride dihydrate, magnesium chloride, and phosphate buffer, with distilled or deionized water serving as the solvent. The formulation also contains carboxymethyl cellulose sodium as a viscosity modifier and methyl paraben as a preservative. The solution was supplied sterile and filtered through a 0.2 µm membrane. The pH was adjusted to 6.8 ± 0.1 at 25 °C.

Communication status was measured before and after immersing the test specimen in artificial saliva. Test pieces were immersed in artificial saliva at 37℃ for 24 h prior to testing. Reading and writing were performed on the IC tags, and the number of test specimens that could communicate at each depth before and after immersion was calculated. The definitions of communication status are as follows. Communication feasible refers to instances in which the IC tag was successfully read by the reader/writer device. In such cases, measurements of the Received Signal Strength Indicator (RSSI) were also obtained. Communication unfeasible refers to instances in which the IC tag could not be read by the reader/writer device at all. Consequently, RSSI could not be obtained under these conditions.

Statistical analysis

In this study, we attempted to assess the association between salivary presence and communication ability using a contingency table. However, since all observations for the "unable to communicate" category were zero, the chi-square test could not be applied. The test requires non-zero expected frequencies in all cells, and the presence of zero values invalidates the calculation of the test statistic. Fisher’s exact test was also considered as an alternative, but it was similarly inapplicable because of the zero-cell issue. Furthermore, due to the complete absence of variation in one category, no other statistical analysis methods could be appropriately applied to this dataset.

## Results

**Figure 3:**
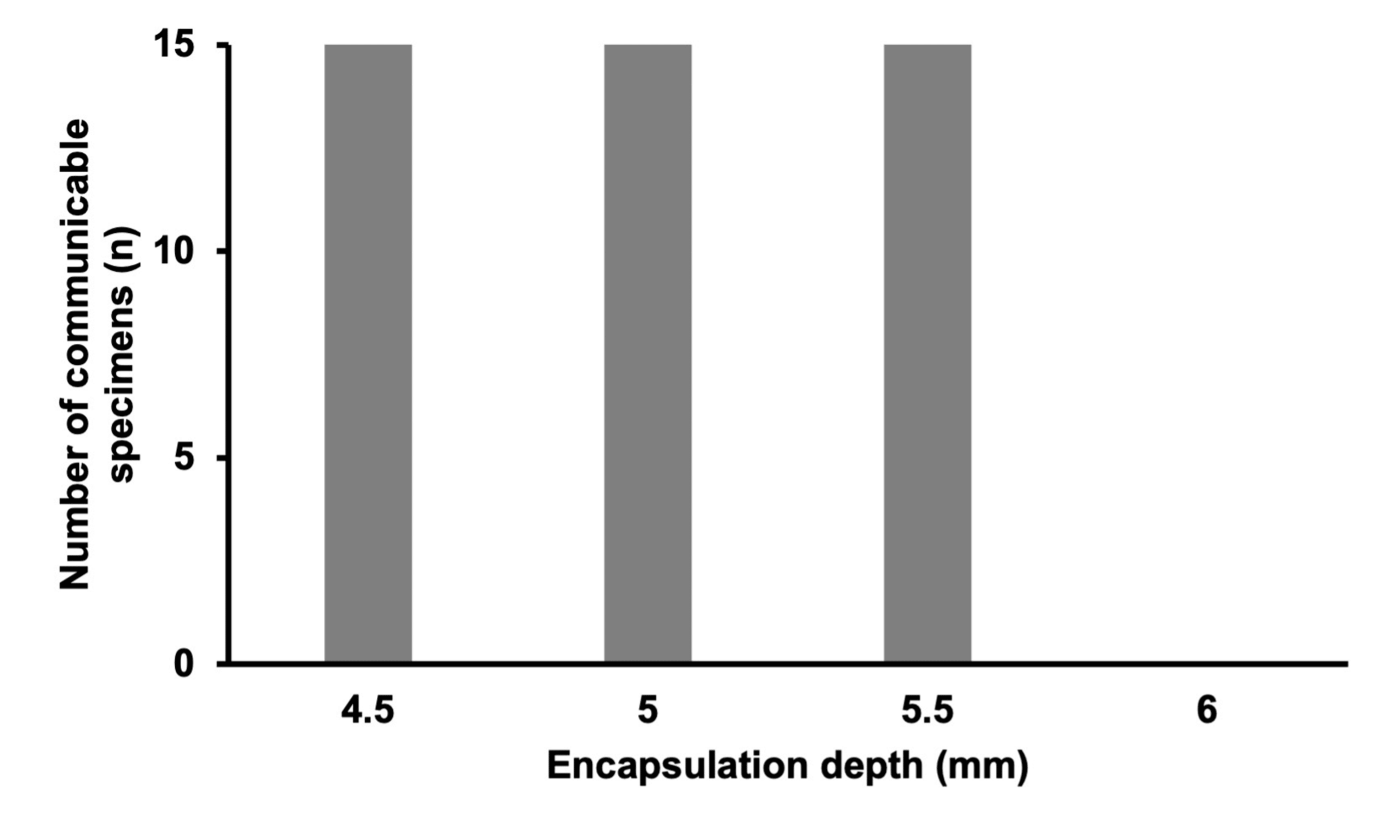
Number of test specimens (n) that were able to communicate in the control group. The data are presented as the number of test specimens (n). Sample size: 4.5 mm (n = 15), 5.0 mm (n = 15), 5.5 mm (n = 15), 6.0 mm (n = 15).

In the control group with the IC tag not encapsulated in dental material, communication was feasible up to a depth of 5.5 mm with all test specimens (n = 15), but communication became unfeasible at 6 mm (Figure 3). At a depth of 5.5 mm, where communication was feasible, RSSI was measured at -60.2 ± 3.1 dBm.

**Figure 4:**
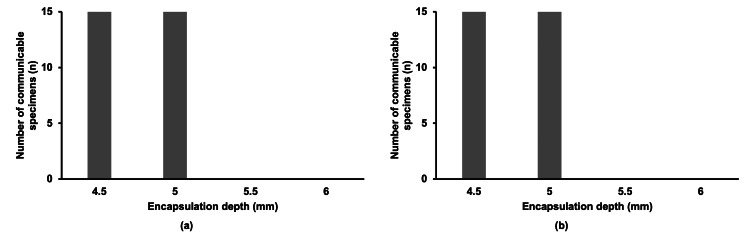
Number of test specimens (n) encapsulated in Unifast III Live Pink that were able to communicate (a) before and (b) after immersion in artificial saliva. The data are presented as the number of test specimens (n). Sample size: 4.5 mm (n = 15), 5.0 mm (n = 15), 5.5 mm (n = 15), 6.0 mm (n = 15).

With Unifast III Live Pink, communication was feasible up to an encapsulation depth of 5.0 mm in all test specimens (n = 15), regardless of immersion in artificial saliva, but communication was unfeasible at depths of 5.5 mm or more (Figure 4). At a depth of 5.0 mm, where communication was feasible, RSSI was measured at -61.8 ± 2.3 dBm before immersion and -61.5 ± 2.1 dBm after immersion.

**Figure 5:**
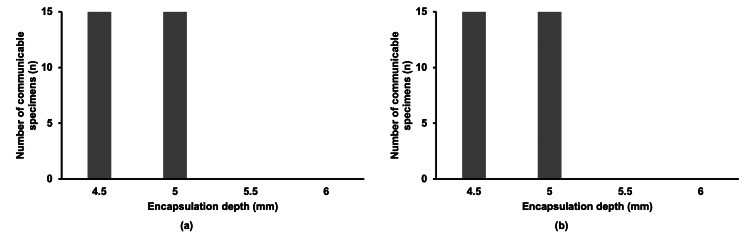
Number of test specimens (n) encapsulated in Unifast III A2 that were able to communicate (a) before and (b) after immersion in artificial saliva. The data are presented as the number of test specimens (n). Sample size: 4.5 mm (n = 15), 5.0 mm (n = 15), 5.5 mm (n = 15), 6.0 mm (n = 15).

Similarly, with Unifast III A2, communication was feasible up to an encapsulation depth of 5.0 mm for all test specimens (n = 15), regardless of immersion in artificial saliva, but communication was unfeasible at depths of 5.5 mm or more (Figure 5). At a depth of 5.0 mm, where communication was feasible, RSSI was measured at -61.0 ± 3.4 dBm before immersion and -61.6 ± 1.6 dBm after immersion.

**Figure 6:**
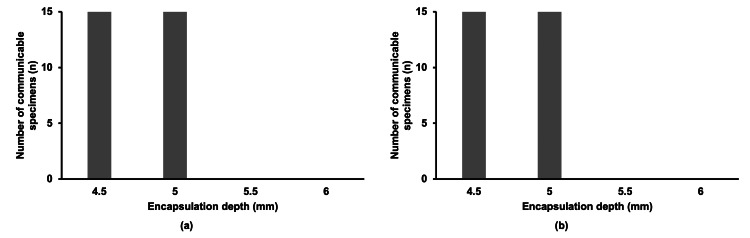
Number of test specimens (n) encapsulated in Clearfil Majesty ES Flow that were able to communicate (a) before and (b) after immersion in artificial saliva. The data are presented as the number of test specimens (n). Sample size: 4.5 mm (n = 15), 5.0 mm (n = 15), 5.5 mm (n = 15), 6.0 mm (n = 15).

With Clearfil Majesty ES Flow, communication was again feasible up to an encapsulation depth of 5.0 mm in all test specimens (n = 15), regardless of immersion in artificial saliva, but communication was unfeasible at depths of 5.5 mm or more (Figure 6). At a depth of 5.0 mm, where communication was feasible, RSSI was measured at -60.3 ± 3.2 dBm before immersion and -60.6 ± 3.1 dBm after immersion.

**Figure 7:**
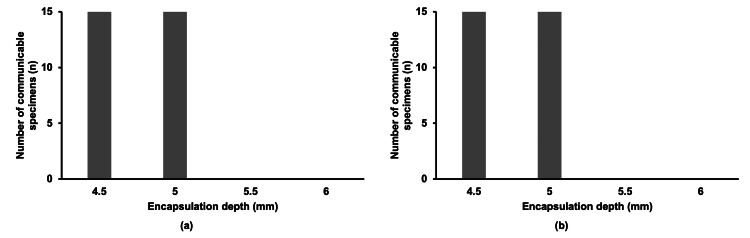
Number of test specimens (n) encapsulated in PRG Protect Seal that were able to communicate (a) before and (b) after immersion in artificial saliva. The data are presented as the number of test specimens (n). Sample size: 4.5 mm (n = 15), 5.0 mm (n = 15), 5.5 mm (n = 15), 6.0 mm (n = 15).

PRG Protect Seal enabled communication up to an encapsulation depth of 5.0 mm in all test specimens (n = 15), regardless of immersion in artificial saliva, but communication was unfeasible at depths of 5.5 mm or more (Figure 7). At a depth of 5.0 mm, where communication was feasible, RSSI was measured at -61.0 ± 3.4 dBm before immersion and -62.2 ± 3.3 dBm after immersion.

## Discussion

To the best of our knowledge, this represents the first study to focus on the possibility of removable dentures as wearable devices. The main findings of this study were that the presence of dental material in the range of 5.0-5.5 mm affects the communication conditions, but immersion in artificial saliva does not.

Fukuda et al. suggested that the communication conditions (damage to IC tags, rain or liquid leakage) affect the ability to utilize IC tags [13]. The present results showed that in the control group, communication was feasible to a depth of 5.5 mm, whereas IC tags encapsulated in various dental materials were capable of communication to a depth of 5.0 mm, regardless of immersion in artificial saliva. In other words, our results suggest that the presence of dental material in the range of 5.0-5.5 mm affects the communication conditions, but immersion in artificial saliva does not. This suggests that using removable dentures as wearable devices may be feasible.

This study aimed to clarify the technological feasibility of applying removable dentures as wearable devices. On the other hand, future studies should also explore their potential for biological monitoring of the oral cavity. With the implementation of removable dentures as wearable devices, various types of information can be obtained from the oral cavity, as these devices can be worn for extended periods. Previous studies have suggested that salivary biomarkers are useful not only for diagnosing and treating oral diseases but also for systemic conditions [18,19]. Therefore, in future studies assessing the long-term biological monitoring potential of salivary biomarkers, the use of removable dentures as wearable devices for continuous saliva monitoring considering clinical situations will be both beneficial and necessary.

Salivary alpha-amylase (sAA) is an enzyme in saliva that has important effects on the formation of dental plaque and caries in the oral cavity [20-22]. However, many cross-sectional studies have shown large variability in the salivary composition of healthy populations [23]. As a result, using saliva as a chair-side screening test for chronic oral diseases such as caries and periodontal disease may be difficult. Longitudinal studies are likely to provide more useful insights into chronic oral diseases than cross-sectional studies [24]. Long-term monitoring using wearable devices would be beneficial for diagnosing chronic oral diseases. In addition, sAA provides a biomarker for the objective assessment of stress [24,25] or pain [26]. Moreover, saliva can be used to detect viruses, fungi, parasites, specific bacteria, and allergic reactions, providing options to non-invasively screen for infections and allergies [18]. Salivary biomarkers have also been shown to provide useful information for the diagnosis and prognosis of various cancers, including oral cancer [27]. Monitoring saliva using wearable devices may contribute to the diagnosis and treatment of various diseases.

Several limitations to this study must be kept in mind. First, this study used only one type of IC tag and only encapsulation depth, the material used for encapsulation, and whether the sample was immersed in artificial saliva were examined. Second, communication distance for the IC tags used in this study was not sufficient to allow use in real-time monitoring. Third, as this study did not involve randomization of measurements or a priori sample size calculation, it may not fully meet the criteria for a powered study. Therefore, the findings should be interpreted as part of an exploratory investigation. Furthermore, there was an inability to perform statistical analysis due to the nature of the data. Specifically, all observations fell into the "able to communicate" category, resulting in zero counts for the "unable to communicate" group. This prevented the application of the chi-square test, as expected frequencies of zero violate its assumptions. Furthermore, no other statistical analysis methods could be appropriately applied given the lack of variation in the data. As part of future considerations, data distribution issues could be mitigated in follow-up designs with larger depth intervals or alternate frequencies. Nevertheless, descriptive statistics clearly indicated that the presence or absence of saliva did not affect communication ability. Further investigation into various communication conditions is warranted to standardize both the encapsulation depth and the materials used for wearable device applications. Such conditions include the use of different types of IC tags, as well as environmental and physiological factors such as temperature fluctuations, pH variation, and dynamic forces associated with chewing or oral movement.

## Conclusions

In summary, this is the first study to investigate the communication environment when applying IC tags to removable dentures, considering the effects of saliva, encapsulation depth, and encapsulation materials, as a step toward applying removable dentures as wearable devices. The present results showed that the encapsulation depth of the IC tag and the dental materials used for encapsulating the IC tag affected communication capability but immersion in artificial saliva did not. Therefore, removable dentures seem to have potential for use as wearable devices.
